# Methyl (1*H*-pyrrol-2-ylcarbonyl­amino)acetate

**DOI:** 10.1107/S1600536808027451

**Published:** 2008-09-06

**Authors:** Gui Hong Tang, Dong Dong Li, Xiang Chao Zeng, Shi Song Dong, Yan Shuang Wang

**Affiliations:** a Department of Chemistry, Jinan University, Guangzhou, Guangdong 510632, People’s Republic of China

## Abstract

In the crystal structure of the title compound, C_8_H_10_N_2_O_3_, mol­ecules are linked by N—H⋯O hydrogen bonds, forming ribbons of centrosymmetric dimers extending along the *c* axis.

## Related literature

For related literature, see: Banwell *et al.* (2006 ▶); Bernstein *et al.* (1995 ▶); Faulkner (2002 ▶); Sosa *et al.* (2002 ▶); Zeng (2006 ▶); Zeng *et al.* (2007 ▶).
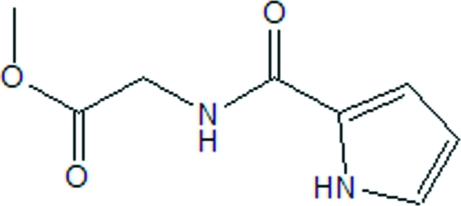


## Experimental

### 

#### Crystal data

C_8_H_10_N_2_O_3_
*M* *_r_* = 182.18
Monoclinic, 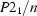

*a* = 11.3398 (19) Å
*b* = 5.0732 (9) Å
*c* = 16.500 (3) Å
β = 108.060 (3)°
*V* = 902.5 (3) Å^3^
*Z* = 4
Mo *K*α radiation
μ = 0.10 mm^−1^
*T* = 173 (2) K
0.48 × 0.41 × 0.21 mm

#### Data collection

Bruker SMART 1K CCD area-detector diffractometer
Absorption correction: multi-scan (*SADABS*; Sheldrick, 1997 ▶) *T*_min_ = 0.952, *T*_max_ = 0.979
4219 measured reflections
1576 independent reflections
1417 reflections with *I* > 2σ(*I*)
*R*_int_ = 0.033

#### Refinement

*R*[*F* ^2^ > 2σ(*F* ^2^)] = 0.052
*wR*(*F* ^2^) = 0.166
*S* = 1.10
1576 reflections
119 parameters
H-atom parameters constrained
Δρ_max_ = 0.21 e Å^−3^
Δρ_min_ = −0.31 e Å^−3^

### 

Data collection: *SMART* (Bruker, 1999 ▶); cell refinement: *SAINT-Plus* (Bruker, 1999 ▶); data reduction: *SAINT-Plus*; program(s) used to solve structure: *SHELXS97* (Sheldrick, 2008 ▶); program(s) used to refine structure: *SHELXL97* (Sheldrick, 2008 ▶); molecular graphics: *SHELXTL* (Sheldrick, 2008 ▶); software used to prepare material for publication: *SHELXTL*.

## Supplementary Material

Crystal structure: contains datablocks I, global. DOI: 10.1107/S1600536808027451/cf2214sup1.cif

Structure factors: contains datablocks I. DOI: 10.1107/S1600536808027451/cf2214Isup2.hkl

Additional supplementary materials:  crystallographic information; 3D view; checkCIF report

**Fig. 1.**
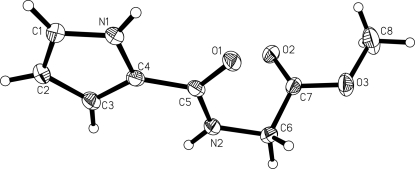
The molecular structure of the title compound, with the atom-numbering scheme. Displacement ellipsoids are drawn at the 30% probability level.

Enhanced figure: interactive version of Fig. 1

## Figures and Tables

**Table 1:** Hydrogen-bond geometry (Å, °)

*D*—H⋯*A*	*D*—H	H⋯*A*	*D*⋯*A*	*D*—H⋯*A*
N1—H1⋯O1^i^	0.88	1.93	2.782 (2)	162
N2—H2⋯O2^ii^	0.88	2.09	2.9372 (19)	161

Symmetry codes: (i) 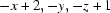; (ii) 
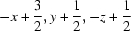
.

**Fig. 2.**
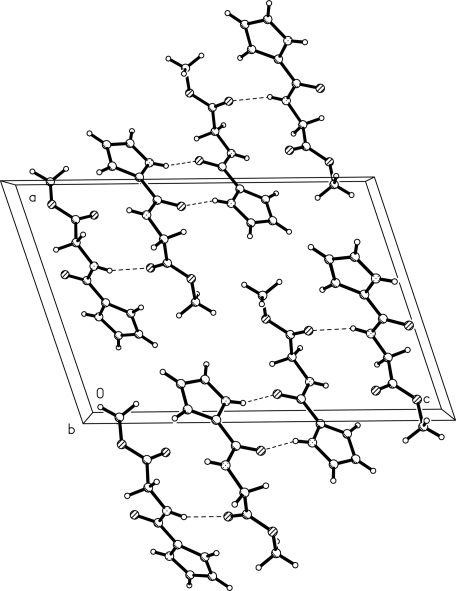
Ribbons of dimers formed by hydrogen bonds (dashed lines).

## supplementary crystallographic information

### Comment

Pyrrole derivatives are well known in many marine organisms (Faulkner, 2002). Some show important bioactivities, such as antitumor activity (Banwell *et al.*, 2006) and protein kinase inhibiting activity (Sosa *et al.*, 2002). This is the reason why they have attracted our interest. This study follows our previous studies on methyl 2-(4,5-dibromo-1*H*-pyrrole-2-carboxamido)propionate (Zeng *et al.*, 2007) and 3-bromo-1-methyl-6,7-dihydropyrrolo[2,3-*c*]azepine- 4,8(1*H*,5*H*)-dione (Zeng, 2006).

In the crystal structure, molecules of the title compound are linked through N1—H1···O1^i^ hydrogen bonds to form centrosymmetric dimers (Fig. 2) of graph-set motif *R*_2_^2^(10) (Bernstein *et al.*, 1995), which are linked by N2—H2···O2^ii^ hydrogen bonds, generating ribbons extending along the *c* axis (also shown in Fig. 2). Bond lengths and angles are unexceptional.

### Experimental

The hydrochloric acid salt of glycine methyl ester (0.63 g, 5 mmol) and 2-trichloroacetylpyrrole (1.06 g, 5 mmol) were added to acetonitrile (12 ml), followed by the dropwise addition of triethylamine (1.4 ml). The mixture was stirred at room temperature for 10 h and then poured into water. After filtration, the precipitate was collected as a yellow solid. The impure product was dissolved in EtOH at room temperature. Light-yellow monoclinic crystals suitable for X-ray analysis (m.p. 420 K, 95.6% yield) grew over a period of one week when the solution was exposed to the air. CH&N elemental analysis. Calc. for C_8_H_10_N_2_O_3_: C 52.74, H 5.53, N 15.38%; found: C 52.78, H 5.59, N 15.49%.

### Refinement

H atoms were positioned geometrically [C—H = 0.99Å for CH_2_, 0.98Å for CH_3_, 0.95Å for CH (aromatic), and N—H = 0.88 Å] and refined using a riding model, with *U*_iso_ = 1.2*U*_eq_ (1.5*U*_eq_ for the methyl group) of the parent atom.

### Crystal data

**Table:**

C_8_H_10_N_2_O_3_	*D*_x_ = 1.341 Mg m^−^^3^
*M**_r_* = 182.18	Melting point: 420 K
Monoclinic, *P*2_1_/*n*	Mo *K*α radiation, λ = 0.71073 Å
*a* = 11.3398 (19) Å	Cell parameters from 3468 reflections
*b* = 5.0732 (9) Å	θ = 2.6–27.0°
*c* = 16.500 (3) Å	µ = 0.10 mm^−^^1^
β = 108.060 (3)°	*T* = 173 K
*V* = 902.5 (3) Å^3^	Block, light yellow
*Z* = 4	0.48 × 0.41 × 0.21 mm
*F*(000) = 384	

### Data collection

**Table:**

Bruker SMART 1K CCD area-detector diffractometer	1576 independent reflections
Radiation source: fine-focus sealed tube	1417 reflections with *I* > 2σ(*I*)
graphite	*R*_int_ = 0.033
φ and ω scans	θ_max_ = 25.0°, θ_min_ = 1.9°
Absorption correction: multi-scan (SADABS; Sheldrick, 1997)	*h* = −13→13
*T*_min_ = 0.952, *T*_max_ = 0.979	*k* = −6→5
4219 measured reflections	*l* = −16→19

### Refinement

**Table:**

Refinement on *F*^2^	Primary atom site location: structure-invariant direct methods
Least-squares matrix: full	Secondary atom site location: difference Fourier map
*R*[*F*^2^ > 2σ(*F*^2^)] = 0.052	Hydrogen site location: inferred from neighbouring sites
*wR*(*F*^2^) = 0.166	H-atom parameters constrained
*S* = 1.10	*w* = 1/[σ^2^(*F*_o_^2^) + (0.1092*P*)^2^ + 0.2414*P*] where *P* = (*F*_o_^2^ + 2*F*_c_^2^)/3
1576 reflections	(Δ/σ)_max_ = 0.001
119 parameters	Δρ_max_ = 0.21 e Å^−^^3^
0 restraints	Δρ_min_ = −0.31 e Å^−^^3^

### Special details

**Table:**

Geometry. All e.s.d.'s (except the e.s.d. in the dihedral angle between two l.s. planes) are estimated using the full covariance matrix. The cell e.s.d.'s are taken into account individually in the estimation of e.s.d.'s in distances, angles and torsion angles; correlations between e.s.d.'s in cell parameters are only used when they are defined by crystal symmetry. An approximate (isotropic) treatment of cell e.s.d.'s is used for estimating e.s.d.'s involving l.s. planes.
Refinement. Refinement of *F*^2^ against ALL reflections. The weighted *R*-factor *wR* and goodness of fit *S* are based on *F*^2^, conventional *R*-factors *R* are based on *F*, with *F* set to zero for negative *F*^2^. The threshold expression of *F*^2^ > σ(*F*^2^) is used only for calculating *R*-factors(gt) *etc*. and is not relevant to the choice of reflections for refinement. *R*-factors based on *F*^2^ are statistically about twice as large as those based on *F*, and *R*- factors based on ALL data will be even larger.

### Fractional atomic coordinates and isotropic or equivalent isotropic displacement parameters (Å^2^)

**Table:**

	*x*	*y*	*z*	*U*_iso_*/*U*_eq_	
O1	0.89632 (13)	0.2572 (2)	0.45357 (8)	0.0387 (4)	
O2	0.63332 (13)	0.2895 (3)	0.31013 (8)	0.0391 (4)	
N2	0.85781 (14)	0.5697 (3)	0.35256 (9)	0.0343 (4)	
H2	0.8777	0.6472	0.3109	0.041*	
O3	0.58657 (13)	0.4945 (3)	0.41547 (9)	0.0513 (5)	
C3	1.06971 (17)	0.3350 (4)	0.29921 (12)	0.0364 (5)	
H3	1.0414	0.4778	0.2608	0.044*	
N1	1.08932 (14)	0.0465 (3)	0.40331 (10)	0.0343 (4)	
H1	1.0773	−0.0386	0.4466	0.041*	
C5	0.92253 (15)	0.3607 (3)	0.39334 (10)	0.0306 (5)	
C6	0.75496 (17)	0.6645 (3)	0.37859 (12)	0.0352 (5)	
H6A	0.7211	0.8266	0.3464	0.042*	
H6B	0.7847	0.7099	0.4400	0.042*	
C7	0.65395 (17)	0.4608 (3)	0.36324 (11)	0.0329 (5)	
C4	1.02305 (17)	0.2601 (3)	0.36367 (11)	0.0313 (5)	
C1	1.17634 (18)	−0.0142 (4)	0.36582 (13)	0.0394 (5)	
H1A	1.2344	−0.1546	0.3817	0.047*	
C2	1.16634 (18)	0.1618 (4)	0.30085 (13)	0.0413 (5)	
H2A	1.2159	0.1652	0.2638	0.050*	
C8	0.4845 (3)	0.3088 (6)	0.40341 (18)	0.0720 (9)	
H8A	0.4282	0.3231	0.3450	0.108*	
H8B	0.4391	0.3494	0.4437	0.108*	
H8C	0.5174	0.1290	0.4135	0.108*	

### Atomic displacement parameters (Å^2^)

**Table:**

	*U*^11^	*U*^22^	*U*^33^	*U*^12^	*U*^13^	*U*^23^
O1	0.0451 (8)	0.0384 (8)	0.0386 (8)	0.0040 (6)	0.0217 (6)	0.0103 (5)
O2	0.0448 (8)	0.0368 (7)	0.0392 (8)	−0.0018 (6)	0.0180 (6)	−0.0094 (6)
N2	0.0403 (9)	0.0297 (8)	0.0388 (9)	0.0010 (6)	0.0207 (7)	0.0065 (6)
O3	0.0508 (9)	0.0644 (10)	0.0499 (9)	−0.0184 (7)	0.0319 (8)	−0.0247 (7)
C3	0.0388 (10)	0.0350 (10)	0.0387 (10)	−0.0025 (8)	0.0166 (8)	0.0063 (8)
N1	0.0376 (9)	0.0304 (8)	0.0385 (9)	−0.0018 (6)	0.0170 (7)	0.0044 (6)
C5	0.0330 (9)	0.0288 (10)	0.0315 (9)	−0.0055 (7)	0.0120 (8)	0.0008 (7)
C6	0.0423 (11)	0.0274 (9)	0.0394 (10)	0.0022 (7)	0.0178 (8)	−0.0003 (7)
C7	0.0383 (10)	0.0322 (9)	0.0304 (9)	0.0046 (7)	0.0141 (8)	−0.0008 (7)
C4	0.0341 (9)	0.0270 (9)	0.0337 (10)	−0.0042 (7)	0.0119 (8)	0.0006 (7)
C1	0.0368 (10)	0.0345 (10)	0.0502 (12)	0.0011 (8)	0.0184 (9)	0.0004 (8)
C2	0.0416 (11)	0.0416 (11)	0.0492 (12)	−0.0029 (8)	0.0265 (9)	0.0022 (9)
C8	0.0677 (16)	0.097 (2)	0.0690 (16)	−0.0400 (15)	0.0468 (14)	−0.0364 (15)

### Geometric parameters (Å, °)

**Table:**

O1—C5	1.239 (2)	N1—H1	0.880
O2—C7	1.204 (2)	C5—C4	1.465 (2)
N2—C5	1.345 (2)	C6—C7	1.505 (3)
N2—C6	1.444 (2)	C6—H6A	0.990
N2—H2	0.880	C6—H6B	0.990
O3—C7	1.328 (2)	C1—C2	1.373 (3)
O3—C8	1.458 (3)	C1—H1A	0.950
C3—C4	1.380 (2)	C2—H2A	0.950
C3—C2	1.398 (3)	C8—H8A	0.980
C3—H3	0.950	C8—H8B	0.980
N1—C1	1.353 (2)	C8—H8C	0.980
N1—C4	1.365 (2)		
			
C5—N2—C6	118.64 (14)	O2—C7—O3	122.96 (17)
C5—N2—H2	120.7	O2—C7—C6	125.73 (17)
C6—N2—H2	120.7	O3—C7—C6	111.30 (15)
C7—O3—C8	114.87 (16)	N1—C4—C3	107.59 (16)
C4—C3—C2	107.31 (17)	N1—C4—C5	119.11 (15)
C4—C3—H3	126.3	C3—C4—C5	133.30 (17)
C2—C3—H3	126.3	N1—C1—C2	108.28 (17)
C1—N1—C4	109.46 (15)	N1—C1—H1A	125.9
C1—N1—H1	125.3	C2—C1—H1A	125.9
C4—N1—H1	125.3	C1—C2—C3	107.37 (17)
O1—C5—N2	120.38 (16)	C1—C2—H2A	126.3
O1—C5—C4	121.72 (16)	C3—C2—H2A	126.3
N2—C5—C4	117.89 (14)	O3—C8—H8A	109.5
N2—C6—C7	111.34 (14)	O3—C8—H8B	109.5
N2—C6—H6A	109.4	H8A—C8—H8B	109.5
C7—C6—H6A	109.4	O3—C8—H8C	109.5
N2—C6—H6B	109.4	H8A—C8—H8C	109.5
C7—C6—H6B	109.4	H8B—C8—H8C	109.5
H6A—C6—H6B	108.0		
			
C6—N2—C5—O1	−2.1 (2)	C2—C3—C4—N1	−0.2 (2)
C6—N2—C5—C4	177.39 (15)	C2—C3—C4—C5	−179.42 (19)
C5—N2—C6—C7	−63.6 (2)	O1—C5—C4—N1	0.2 (3)
C8—O3—C7—O2	−0.4 (3)	N2—C5—C4—N1	−179.30 (14)
C8—O3—C7—C6	178.56 (19)	O1—C5—C4—C3	179.36 (19)
N2—C6—C7—O2	−26.5 (3)	N2—C5—C4—C3	−0.1 (3)
N2—C6—C7—O3	154.55 (16)	C4—N1—C1—C2	−0.1 (2)
C1—N1—C4—C3	0.2 (2)	N1—C1—C2—C3	0.0 (2)
C1—N1—C4—C5	179.57 (16)	C4—C3—C2—C1	0.1 (2)

### Hydrogen-bond geometry (Å, °)

**Table:**

*D*—H···*A*	*D*—H	H···*A*	*D*···*A*	*D*—H···*A*
N1—H1···O1^i^	0.88	1.93	2.782 (2)	162.
N2—H2···O2^ii^	0.88	2.09	2.9372 (19)	161.

Symmetry codes: (i) −*x*+2, −*y*, −*z*+1; (ii) −*x*+3/2, *y*+1/2, −*z*+1/2.
